# Views on sharing mental health data for research purposes: qualitative analysis of interviews with people with mental illness

**DOI:** 10.1186/s12910-023-00961-6

**Published:** 2023-11-14

**Authors:** Emily Watson, Sue Fletcher-Watson, Elizabeth Joy Kirkham

**Affiliations:** 1 University of Edinburgh Medical School, Edinburgh, UK; 2 Centre for Clinical Brain Sciences, University of Edinburgh, Edinburgh, UK; 3 Clinical Psychology, School of Health in Social Science, University of Edinburgh, Edinburgh, UK; 4 Medical School, Teviot Place, Edinburgh, EH8 9AG UK

**Keywords:** Mental health, Health data, Patient perspectives, Interview, Mental illness, Qualitative, Electronic health records

## Abstract

**Background:**

Improving the ways in which routinely-collected mental health data are shared could facilitate substantial advances in research and treatment. However, this process should only be undertaken in partnership with those who provide such data. Despite relatively widespread investigation of public perspectives on health data sharing more generally, there is a lack of research on the views of people with mental illness.

**Methods:**

Twelve people with lived experience of mental illness took part in semi-structured interviews via online video software. Participants had experience of a broad range of mental health conditions including anxiety, depression, schizophrenia, eating disorders and addiction. Interview questions sought to establish how participants felt about the use of routinely-collected health data for research purposes, covering different types of health data, what health data should be used for, and any concerns around its use.

**Results:**

Thematic analysis identified four overarching themes: benefits of sharing mental health data, concerns about sharing mental health data, safeguards, and data types. Participants were clear that health data sharing should facilitate improved scientific knowledge and better treatments for mental illness. There were concerns that data misuse could become another way in which individuals and society discriminate against people with mental illness, for example through insurance premiums or employment decisions. Despite this there was a generally positive attitude to sharing mental health data as long as appropriate safeguards were in place.

**Conclusions:**

There was notable strength of feeling across participants that more should be done to reduce the suffering caused by mental illness, and that this could be partly facilitated by well-managed sharing of health data. The mental health research community could build on this generally positive attitude to mental health data sharing by following rigorous best practice tailored to the specific concerns of people with mental illness.

**Supplementary Information:**

The online version contains supplementary material available at 10.1186/s12910-023-00961-6.

## Background

Large data sets, such as those generated from the routinely-collected data in health records, are becoming increasingly important for public health, contributing to improved service implementation, earlier disease prevention and treatment advances [1–3]. The term “routinely-collected data” has been defined in different ways, but in general it refers to data that were originally collected for a purpose other than research [4]. This could include but is not limited to health records [5], Census information [6], social media posts [7], and information held by government departments [8]. The present work focuses primarily, though not exclusively, on health records as a source of routinely-collected data. In relation to mental health, routinely-collected data has been used for a range of purposes, including developing more effective ways to identify suicide risk [9], examining the effect of neighbourhood regeneration on mental health [10], and identifying the extent to which antipsychotic medication is being prescribed for autistic children [11].

Research which uses routinely-collected data has advantages and disadvantages when compared to the more traditional research approach of recruiting individual participants to a specific study. When working with traditional methods researchers have more control and flexibility over the types of data that are collected and the research questions they can ask [12]. On the other hand, researchers using routinely-collected data can often access very large samples which facilitate discovery of small statistical effects which would be difficult to find in smaller samples [8, 12]. Furthermore, routinely-collected health data can theoretically include all individuals who receive health care, as they do not rely on patients’ ability to contribute their own time and effort to research studies [13–15]. This can facilitate data samples that are more representative of the target population [16]. Both traditional methods and routinely-collected data are important for mental health research.

Whilst routinely-collected data can have major benefits for health research, they should only be used in a manner that is acceptable to the people whose data are being studied. In the case of health records, this may be the general public, or the individuals living with the condition(s) being investigated. The dangers of attempting to use such data without broad societal support were illustrated by the public failure of the “care.data” scheme. This was an English initiative designed to gather data from health records which was suspended and then scrapped following widespread public and professional outcry [17]. Carter and her colleagues argue that the scheme violated two of the key components involved in the use of routinely-collected data: trust, and confidence that the data would be used for public good. By contrast, successful UK initiatives such as the Case Register Interactive Search tool (CRIS) and the Secure Anonymised Data Linkage (SAIL) databank prioritised patient and public involvement in both their inception and their ongoing operations [18, 19].

Previous work on this topic has found relatively high levels of public support for health data sharing (73–93% positive; 20, 21–23]. However, this support is somewhat conditional, such that individuals typically want their data to be handled by an organisation they trust and used for the public good rather than for profit [24, 25]. Research also suggests that people’s views vary according to the perceived sensitivity of the data, with mental health data cited as an example of sensitive health data [26, 27]. The extent to which people with mental illness agree with this perception of mental health data as especially sensitive, or how they feel more broadly about sharing mental health data, remains unclear [28]. This is because the views of people with mental illness are largely missing from literature on health data sharing [23, 28]. For example, none of the 25 studies emerging from Aitken and colleagues’ systematic literature review on health data sharing had explicitly recruited people with mental illness [25].

The small amount of prior research which has focused on the preferences of people with mental illness found that factors which influence willingness to share health data include their prior experiences with health care services [23, 29, 30], stigma and the perceived risk of discrimination [31], and, similar to the wider general public, their trust in the organisation accessing the data [32]. There is also early evidence to suggest that people with mental illness may actually be more willing than those without to share their health data for research purposes [24], at least once other factors such as lower overall satisfaction with healthcare have been accounted for [23]. This may be due to a desire to help other people with the same health conditions [23, 28]. However, this finding of increased willingness to share such data is not universal [33].

At present, researchers’ access to routinely-collected mental health data is heavily limited, in part due to concerns around the presumed sensitivity of such data [34]. However, it remains unclear whether this approach reflects the preferences of people with mental illness, due to a lack of research on the topic [24]. Much of the research which does exist in this field focuses on survey data [20, 23, 24], and is therefore better suited to identifying what people think rather than why they think it. Understanding the details and rationale behind people’s perspectives is necessary if efforts to increase the use of routinely-collected mental health data are to succeed [17]. In light of this, we conducted semi-structured interviews with 12 people with a range of mental health conditions to explore their perspectives on the use of routinely-collected mental health data for research purposes. The aim of the research was to identify how people with mental illness feel about mental health data sharing, and to understand why they felt this way.

## Methods

### Recruitment

Prior to the present interview study, our research team conducted a UK-wide online survey examining perspectives on mental health data sharing. This project is described in detail elsewhere [23, 35]. Briefly, the survey was completed by approximately 1500 participants who responded to advertisements publicised via social media, posters, a science festival, and relevant research and clinical networks. Information on participant mental health was collected during the survey using a list of conditions that had been reviewed by clinicians, with an option for a free text entry if a specific condition was not covered. Individuals who took part in the survey were asked to leave their email address if they were interested in taking part in an interview on the same topic (mental health data sharing).

This list of email addresses was used to invite people with mental health conditions to take part in the present interview study. We aimed to make the interview sample as representative of the UK population as possible by prioritising invitations to demographic groups that were under-represented in the survey (men, people from minoritised ethnicities, and people who had not attended university). Prior to issuing invitations, we used the survey responses to identify potential participants with a diverse range of mental health conditions. We also sought specifically to invite participants from the minority who had responded “no” when the survey asked if they would share their mental health data for research purposes. This was challenging because the vast majority of survey participants (89.7%) had responded “yes” to this question, and those who didn’t often also did not consent to be followed up for this study.

### Participants

Interview participants demographics, presented in Table 1, were extracted from their aforementioned survey responses. The demographics of the interview sample were similar to the demographics of the survey sample [23], especially with respect to ethnicity and location within the UK. However, the interview sample contained a higher portion of male participants (50%) than the survey sample (33%), and as such more closely represented the gender distribution of the UK as a whole. While both the survey and interview samples contained a high proportion of individuals who had attended university, this was especially high in the interview sample (interview: 92%, survey: 60%). Participants’ experiences of mental illness were extracted from the survey data and confirmed at the beginning of the interview.

**Table 1:** Participant Demographics

Participant	Gender	Age range^a^	Ethnicity	Location	Highest Level of Education	Mental Illness Experience
1	Female	30–40	White	England	Postgraduate degree	Anxiety (primary*), depression
2	Male	40–50	White	Scotland	Undergraduate degree	Schizophrenia (primary), addiction
3	Female	40–50	Mixed/multiple ethnic groups	Wales	Undergraduate degree	Bipolar disorder (primary), depression, anxiety (participant added PTSD** during interview)
4	Female	40–50	White	Scotland	Undergraduate degree	Anxiety, PTSD** (primary), depression
5	Male	40–50	White	Scotland	Postgraduate degree	Addiction, anxiety, depression, autism*** (primary)
6	Female	40–50	White	England	Postgraduate degree	Anxiety, bipolar disorder (had diagnosis removed), depression (no primary condition chosen)
7	Female	40–50	White	England	A-level	Depression (primary), anxiety, eating disorder, self-harm
8	Male	40–50	White	England	Undergraduate degree	Anxiety, depression (primary), self-harm
9	Male	30–40	White	England	Postgraduate degree	Anxiety, depression (primary), eating disorder, self-harm
10	Male	30–40	White	England	Postgraduate degree	Anxiety, depression (primary)
11	Non-binary	20–30	White	Scotland	Undergraduate degree	Anxiety (primary), depression
12	Female	30–40	White	England	Postgraduate degree	Anxiety, depression (primary)

*Note.*^a^Age (in years) is provided as a range to protect participant anonymity. *“Primary” mental health condition was defined by the respondent as the condition which had the biggest impact on the participant’s daily life. **PTSD = post-traumatic stress disorder. ***Autism is not a mental health condition but is included here in cases where participants chose to add autism themselves when asked to report their mental health conditions. As we did not explicitly ask about autism, it is not possible to determine whether or not other participants were also autistic

### Positionality statement

This paper is written by three white women from the UK, two of whom are researchers and one of whom is a medical student at the time of writing. The interviewer is a female researcher who has lived experience of mental illness, a PhD in Psychology, and professional expertise in working with mental health data. She is part of a university research group which uses Big Data to answer questions about mental health. The person who analysed the data is a female fifth-year medical student (at the time of writing) who has completed a placement in psychiatry. She has previous experience in qualitative research during her intercalated BSc year and a BSc in Physical Activity for Health. The student who completed the analysis was supervised by the researcher who conducted the interviews.

Interviews were arranged by email. The participants did not have any other contact with the interviewing researcher prior to the interviews themselves. The participant information sheet stated that individuals aged 16 or over who had lived experience of mental illness and experience of using the NHS (for any reason) were “ invited to take part in an interview about [their] views on sharing health data for research purposes”. The University which hosted the research was named on the information sheet. The information sheet did not contain details about the interviewer’s background, but it could be deduced that she was a woman with a PhD.

### Interview guide

The interview guide was developed through discussion within the research team, including an advisory group of community representatives (with lived experience of mental illness and working in mental health services) around the question “How do people feel about data sharing?”. An interview script from a previous qualitative study conducted by SFW’s team was used for guidance during the design phase [36]. The interview guide was semi structured, and the interviewer asked complementary questions if she deemed it appropriate. The interviewer engaged in a practice interview with Suzy Syrett, a peer researcher who has lived experience of mental illness and expertise in conducting research interviews. The full interview guide is available in the supplementary material.

### Procedure

Ethical approval for this study was provided by the Department of Clinical and Health Psychology Ethics Research Panel, University of Edinburgh, ref STAFF147. The research was performed in accordance with the ethical principles of the Declaration of Helsinki. All participants were provided with an information sheet and completed an informed consent form prior to taking part in the research. Semi-structured interviews were carried out online via Skype between July and November 2019. The researcher continued to conduct interviews until the point at which perceived data saturation was reached [37, 38]: i.e. there was the subjective impression that each new participant was largely presenting perspectives already represented in the data. No other individuals were present at the time of interview. Before asking the pre-determined questions, the interviewer allowed time for the participant to become comfortable. It has been shown that allowing for this familiarity to develop fosters in-depth discussions [39, 40]. During this time the interviewer described the terminology that she intended to use during the interview (i.e. “mental illness”) and checked whether the participant was comfortable with this terminology, or if they would prefer that the interviewer used different wording. The interviewer also spoke informally with the participant to check how they were in themselves and to reiterate the topic of the interview. Participants were asked if they would like to go through the information sheet (which they had read previously) with the interviewer, and if they had any questions about the interview process. This allowed the interviewer and participant to confirm that informed consent remained in place, and that the participant’s capacity to consent had not changed since they agreed to take part in the research. Once this had been established, the interviewer checked that the participant was happy for the subsequent conversation to be recorded. The interviewer then turned on the recording device and began the interview.

Interviews were recorded using the MP3 Skype recorder, which recorded only audio. The researcher who conducted the interviews transcribed five of the interviews and the remainder were transcribed by a paid professional transcriber. Due to a technical error, one interview was not recorded. In this case the interviewer’s notes were used instead. All identifiable information was removed from transcripts.

### Data analysis

**Fig. 1:**
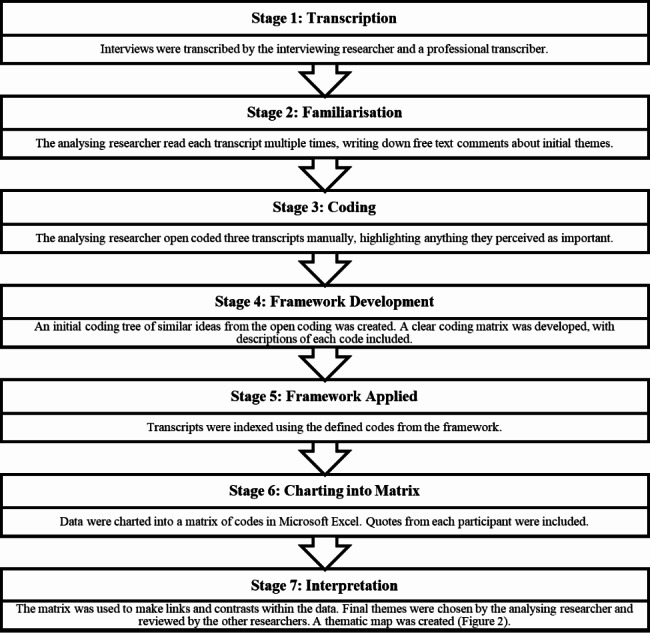
The Framework Method (Amended from Gale, Heath, Cameron, Rashid & Redwood, 2013)

**Fig. 2:**
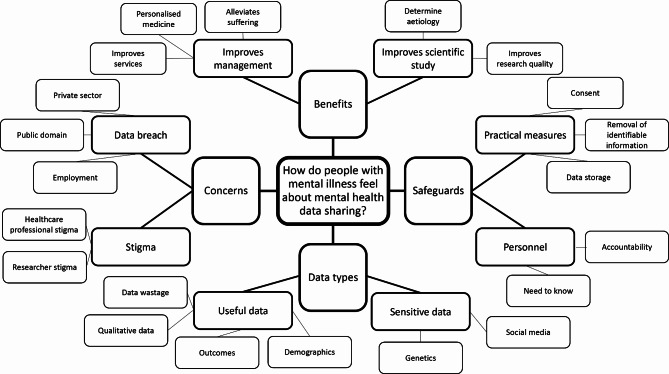
Hierarchical Thematic Map

The structure of the analytical process was informed by the Framework Method [41], and both inductive and deductive coding were employed. Themes were not pre-planned and were derived from the dataset, but they were shaped by the research question: namely to discover how people with mental illness felt about mental health data sharing. The analysing researcher began by coding three transcripts, then using this information to develop a framework of codes (matrix). This framework matrix was then used to code the remaining transcripts (Fig. 1). After all transcripts had been coded, the analysing researcher used the completed matrix to interpret the data and generate themes. The themes were then reviewed by the other researchers. Following analysis, a thematic tree was created (Fig. 2).

## Results

Twelve interviews were conducted with people who had personal experience of mental illness. The mean interview length was 36 min, with the shortest interview lasting 15 min and the longest lasting 77 min.

Through framework analysis, four top level themes and eight subthemes were identified (Fig. 2). Details of the themes are included in Table 2 alongside illustrative quotes. Most participants were positive about data sharing for mental health research purposes, with one person stating *“I am overwhelmingly I think, positive about data sharing, including with mental health*”. This generally positive attitude was accompanied by acknowledgement of the ways in which legitimate and illegitimate data access could be used to discriminate against people with mental illness, and a desire to mitigate against this.

**Table 2:** Themes and subthemes derived from analysis of interview data

Theme/ Subtheme	Quote
**BENEFITS OF SHARING MENTAL HEALTH DATA**	
***1. Delivering improvements for others with mental illness***	
Determine Aetiology	“if in the future, we realise ‘hey there are some other signs and symptoms that perhaps are red flags or signals’”
Improves Research Quality	“Where the research is strengthened by the data and where the study is strengthened by the data”
	“a lot of like statistical power…to pick out…if you have a particular er pre- pre- sort of disposition to developing something if you suffer from this illness”
Alleviate Suffering	“If there was a button I could push and take bipolar disorder away from the world I would. And, the only way we can do that is by letting people research the illness itself”
	“I don’t mind researchers having that information because I strongly believe in helping”
***Subtheme 2: Improving treatment and management of mental health conditions***	
Personalised Medicine	“moving beyond a one size fits all approach”
	“way of better tailoring treatments to the patient, so … yeah. Something called personal treatment or personalised medicine”
Improves Services	“the NHS finally gets the funding it needs and deserves and that universal health care free at the point of need”
	“it might might do more to er, protect it [the NHS] into the future if people are seeing the benefits of that sort of centralised information and erm care”
	“I do feel that the current em … sort of mental health service offerings are somewhat restrictive”
**CONCERNS ABOUT SHARING MENTAL HEALTH DATA**	
***Subtheme 3: Stigma***	
Researcher Stigma	“… I think there’s- there’s more danger in that, erm as in, you know you’re making a judgement rather than actually, erm, sort of asking the question of me myself”
	“dangerous world views on certain mental health issues …I can’t think of the right words but like a damaging world view, or personal view on certain mental health issues”
Healthcare Professional Stigma	“the term is “diagnostic overshadowing” but if if you go somewhere, I mean suppose I rocked up to A & E one evening complaining of dreadful stomach pains… if if it flashed up and said “well this person is a long term mental health service user”, that wouldn’t get taken seriously”
***Subtheme 4: Data misuse and data breaches***	
Private Sector	“I guess if there was personal data in there they could, you know er a random company could er contact you or, you know… maybe someone you know works in a company
	“scare mongering about in the future we might have a privatised NHS um insurance companies might get that kind of information, you might be excluded from reasonably priced insurance”
Public Domain	“If it got leaked to my ex-husband. And he went for sole custody of my daughter, that is the worst case scenario I could imagine”
	“the worst case possible, if my full NHS record was available online for anyone to download and read”
Employment	“employer discrimination I can see being a worry”
	“I think that information being shared with people like your employer without your consent…that kind of information wouldn’t be used to either … make somebody redundant or to put them through sanctions of some description or demotion or something like that”
**SAFEGUARDS**	
***Subtheme 5: Personnel***	
Need to Know	“Keeping the number of people that actually see it reduced to the people that actually need to see it”
	“well you know like researchers have access to the data they need and nothing more”
Accountability	“it would be known that they have seen it and therefore any issues could be tracked back to them”
***Subtheme 6: Practical Measures***	
Removal of Identifiable Information	“a separate identifier so there’s absolutely no need for, there’s no need for names”
	“all the information was anonymised and it couldn’t be traced back to… me”
Consent	“I think if information is going to get passed on, I would like to say yes or no…knowing that you can withdraw at any time …Em … and as far as possible I would like to give consent for those kinds of things on an individual, case by case basis”
Data Storage	“how the data is stored and obviously there is GDPR rules and universities have their rules”
	“I think if my data could be used in a, a more secure way then I would be very happy with that”
	“maybe you need a password to download it”
**DATA TYPES**	
***Subtheme 7: Useful Data***	
Qualitative Data	“People’s opinions on the service that they’ve received I suppose”
	“look at how doctors perceive different patients, different conditions, what ways are they talking about the patients”
Demographics	“It might help inform sort of realising that there is sort of major socio-economic issues in this particular health issue”
Predictors and Outcomes	“things have got so bad that you know you’ve taken your own life. Erm, and try and- you know, the kind of things that have led up to that I guess, those are, pretty important”
	“I think trying to assess which kind of treatments work in what situation”
***Subtheme 8: Sensitive Data***	
Social Media	“access to use social media stuff I think as well can be a bit personal”
Genetics	“I guess if you have genetic material erm, or genetic information, and you’re looking for one thing and you find something else, erm, that can have an impact on someone’s life”
Mental and Physical Health	“they are actually connected”
	“my physical health data isn’t anything like as personal”
	“I think there needs to be more research looking at how they interact, physical and mental health”

### Theme 1: benefits of sharing mental health data

#### Subtheme 1: delivering improvements for others with mental illness

There was an overarching perception that sharing data could drive research progress, which in turn could reduce the suffering of other people with mental health conditions. One participant said, *“if there was a button I could push and take bipolar disorder away from the world I would”*. Several participants described the potential ways increased data sharing could support scientific research, using phrases such as “statistical power” in reference to the ability to uncover more effects with larger sample sizes. One participant stated *“I don’t mind researchers having that information because I strongly believe in helping”.* Participants talked about the role of research in uncovering the aetiology of mental health conditions, and valued the role of data in supporting robust research.

#### Subtheme 2: improving treatment and management of mental health conditions

Multiple participants described negative personal experiences of UK mental health services and their accessibility. They referred to UK mental health services as “*restrictive*”, “*shocking*” and “*frustrating*”, with one participant stating they hoped that *“the NHS finally gets the funding it needs”*. Within this context participants highlighted the potential for data sharing to facilitate improved mental health services. They also felt that sharing mental health data could lead to better treatments for mental ill health, with some discussing the concept of *“personalised medicine,”* in which treatments are tailored to a specific patient or group of patients. The adoption of a personalised medicine approach has helped to reduce mortality in cancer and heart disease, yet remains absent from mental health care, in part due to an historical lack of large mental health datasets [42]. The value of personalised medicine for mental health was highlighted by several of the participants’ own experiences of “trial and error”, with one individual expressing frustration about being placed on eleven different medications before finding one that worked.

### Theme 2: concerns about sharing mental health data

#### Subtheme 3: stigma

Concerns about being subject to stigma from the researchers who would potentially have access to their data came up several times, with one participant worrying that researchers may have *“dangerous world views on certain mental health issues.”* Participants also noted that greater access to mental health data could allow for increased discrimination from healthcare professionals. For example, one participant discussed the potential for increased data sharing to lead to more *“diagnostic overshadowing”.* Diagnostic overshadowing describes a phenomenon in which patients with mental illness are less likely to receive appropriate and timely care for a physical health condition because clinicians incorrectly attribute their symptoms to mental illness [43].

#### Subtheme 4: data misuse and data breaches

Many participants discussed concerns about data breaches, ranging from data misuse by private sector companies to impacts on their personal life. They expressed fear of being *“excluded from reasonably priced insurance”*, worries about *“employer discrimination”* and even losing child custody. It is important to note that these concerns were not necessarily focused on the risk of a data breach per se, but the risk of a data breach within a society that already discriminates against people with mental illness. For example, one participant discussed his experience of being denied life insurance after he bought his house, noting that “[mental health] seems to be the most strictly clamped down [on by insurance companies] out of anything else”, more so than physical risk factors such as smoking. He added, “it makes you feel uneasy that you are being financially disadvantaged because of some [mental illness] symptoms that you have had”.

### Theme 3: safeguards

#### Subtheme 5: personnel

Participants believed that researchers should have access to their mental health data on a *“need to know”* basis and that they should only be able to access specific information relevant to their research. Further, the concept of researcher accountability and institutional oversight appeared important, with one individual stating they would like any issues to be *“tracked back”* to the researcher.

#### Subtheme 6: practical measures

Overall participants appeared to have a high degree of understanding of safeguards in place for maintaining confidentiality within research. This may be due to the high proportion of participants with postgraduate degrees and relevant professional experience in the current sample. In fact, one individual said, *“I am a fairly knowledgeable member of the public, so I don’t know if I am typical in that sense”*. Most participants were happy for their data to be shared if all identifiable information was removed. However, some participants voiced concerns about being identified even if “*pseudonyms*” were used.

The principle of informed consent seemed very important to participants with regards to data sharing, with one participant saying, “*I think if information is going to get passed on, I would like to say yes or no…knowing that you can withdraw at any time.”* However, there were inconsistencies between participants with regards to what they considered to be appropriate consent processes. One individual wanted to give consent on a *“case by case”* basis, whilst others didn’t feel as strongly.

### Theme 4: data types

#### Subtheme 7: useful data

A few participants identified that demographic data about people with mental health conditions might be particularly useful in research, with one individual stating that acquisition of such data *“might help inform sort of realising that there is sort of major socio-economic issues in this particular health issue”.* Furthermore, qualitative data such as *“opinions on the service”* and how doctors are *“talking about the patients”* were deemed particularly relevant to research. Several participants also mentioned the potential value of collecting data on factors associated with suicide. One participant commented on making the most of the existing data within NHS health records, *“I think if my data could be used in a, a more secure way then I would be very happy with that. Otherwise, it’s just sitting there, and people could be benefitting from that”*.

#### Subtheme 8: sensitive Data

Participants expressed opinions about certain data types being particularly sensitive. Some referred to social media data as being *“a bit personal”.* Notably, many participants identified genetic data as being particularly sensitive, raising concerns such as the possibility of “eugenics”. As aforementioned, the risk of identification in relation to mental health data worried participants. Participants held contrasting views about the relative sensitivities of physical and mental health data. Some participants believed that they should be considered “*equal*”, whilst others made it clear they thought that mental health data were more personal.

## Discussion

This research used interviews and thematic analysis to investigate how people with mental illness feel about sharing their mental health data for research purposes. Broadly speaking, participants perceived mental health data sharing as a resource that could be used to improve the lives of the wider population of people with mental illness. In particular, there was a focus on the potential for mental health data to facilitate scientific understanding, better treatment options, and improved services. Concerns were often connected to the potential for increased data access (whether sanctioned or illicit) to facilitate discrimination from researchers, healthcare professionals, and wider society (e.g. insurance companies). Participants generally advocated for the use of appropriate safeguards, such as de-identification or restrictions on who could access the data. There was an appreciation that a broad range of routinely-collected data could be useful for mental health research (e.g. demographic data, language used by health professionals), but the perceived sensitivity of these different types of data varied across participants.

The present finding that mental health data should be used for the “greater good” of people with mental illness is similar to the wider literature on the broader public’s perspective on sharing routinely-collected health data for research purposes [25, 44]. Previous work has found that support for data sharing is linked to the perceived benefits to community, public and science [45]. Given the strength of feeling from many of the participants in the present research, it is possible that this altruistic approach to sharing health data may be particularly salient amongst people with mental illness (though we acknowledge that the limited representativeness of the present sample precludes firm assumptions of this nature). There was an overarching sense from the interviews that living with mental illness is extremely difficult, and many participants were motivated by a desire to prevent others going through the same experiences they had. Increased data sharing was perceived as one way to achieve this goal.

A core theme of the present research was the backdrop of stigma and discrimination against people with mental illness in society. Some, though not all, participants perceived mental health data as more sensitive than physical health data, and those that did often couched their concerns in the context of the risk of experiencing more discrimination, whether from researchers, healthcare professionals, private companies, or wider society [43, 46]. Research into perspectives on routinely-collected data often refers to mental health data as “sensitive” [12], but the reasons behind this assumption have been relatively under-explored [23]. The present study adds more nuance to this topic and reiterates the chilling effect of stigma on scientific progress. In our co-produced guidance for mental health data science we highlighted the need for researchers to recognise the wider context in which their work takes place and take a proactive approach to reducing stigma within the field [16, 47].

Participants highlighted various ways to mitigate the risks associated with sharing mental health data in a potentially discriminatory society. This included the need for particular care around de-identification [34, 48], and a reminder that researchers should consider the various ways individuals could be identified from pseudonymous data (e.g. the combination of information across multiple fields could be used to identify an individual). In some cases participants also highlighted the need for control over their data in the form of consent mechanisms. The topic of consent is arguably one of the most challenging within health data sharing. Whilst members of the public sometimes express that they want to provide consent on a case-by-case basis, this can be impractical and work against some of the aforementioned benefits of data sharing, such as the unique opportunities afforded by large sample sizes. Notably, Aitken and colleagues [25] found in their systematic review that, though their initial preference may be for opt-in consent, participants typically move away from this model of consent following discussion of its implications. Nevertheless, it is important that policy decisions acknowledge the broad range of views surrounding the topic of consent. One option proposed by Jones and colleagues [24] would be to manage research access to NHS health data on an opt-out basis, with participants given the option to opt-out on the basis of whether the data would be used for clinical or research purposes, and whether or not it would be potentially identifiable. Future work should build on this nuanced approach to the question of consent, for example by exploring how people with mental illness feel about providing advanced and/or retrospective consent for research access to their health records.

Although the aim of this research was to examine perspectives on mental health data sharing, it would be remiss not to discuss participants’ difficulties accessing adequate care from UK mental health services. It is important to note that these interviews were conducted in 2019, prior to the onset of the Covid-19 pandemic. This highlights that whilst the pandemic has increased the burden on mental health services [49], these services were already struggling to meet people’s needs prior to the pandemic [23]. The myriad challenges faced by under-funded mental health services in the UK are extensive and have been discussed elsewhere [35, 50–52]. However, one lesser-known impact of under-resourced mental health services is the effect on research and development. Many of the UK government’s priorities, such as the development of digital infrastructure, embedding of inclusive practices, and prioritisation of mental health [53–56], are held back by struggling services’ inability to facilitate them. Practical ways in which these goals are being hindered include reliance on over-stretched NHS staff to record high quality data [57], limited structural capacity to facilitate the data flow between the NHS and trusted researchers [34, 57], and a low level of satisfaction with the NHS [58] which itself has been associated with reduced willingness to share mental health data [23]. Thus, investing in mental health services would likely have the added benefit of facilitating faster scientific progress in the field of mental health.

### Strengths and limitations

A key strength of this study is that the sample was made up of individuals with mental health conditions, whose opinions are rarely prioritised in research on health data sharing. Importantly, individuals had experience with a variety of mental health conditions, including schizophrenia, bipolar disorder and depression. The results of qualitative research are not designed to be applicable to the entire population. Nonetheless, a limitation of this study is the lack of racial diversity within the dataset. Eleven of the twelve participants were white. This is especially pertinent to the topic of mental health data sharing, given the associations between minoritised populations and mental health, and the many historical and recent examples of racism within health research [59–63, 57–60, 56]. In addition, the current interview sample was highly educated, which may have influenced their knowledge of and attitudes towards data sharing. Furthermore, as noted in the positionality statement, the researchers are similar to most of the interview participants in terms of their ethnicity and educational experience. This could have limited the extent to which the research project was able to highlight perspectives outside of the contributors’ shared frame of reference.

It is possible that self-selection bias was present. It is feasible that individuals who volunteered to participate in an interview on the topic of mental health data hold more positive views towards mental health data sharing than those with mental illness who did not volunteer. As described above, participants were invited to take part if they had agreed to be contacted for this purpose following completion of our previous survey on health data sharing [23]. When recruiting for the present study we actively tried to invite individuals who had previously responded in the survey that “no”, they were unwilling to share their mental health data for research purposes. However, given that only 10.3% of the full survey sample selected this option, this was challenging, and as a result only two of the final interview sample had responded “no”. Despite this, the participants did vary on a more nuanced survey question in which they used a 5-point Likert scale to indicate how likely they would be to share mental health data, with four of the 12 participants responding “very unlikely” or “unlikely”.

### Future directions

[63]As identified, fear of stigma is a major concern with regards to mental health data sharing. Future research could examine whether interventions designed to reduce mental health stigma are predictive of changes in attitudes to mental health data sharing. Additional findings from this project suggest that knowledge about data safeguards is important to individuals. As such, researchers should endeavour to make these processes transparent, such as by following published guidance on good practice in mental health data sharing [47].

In light of the aforementioned limited representativeness of the current sample, it is important that future research includes more perspectives from those who are less commonly included in work of this nature. In particular, researchers need to do more to actively reach out to people from Black communities [59], people with co-morbid mental and physical health conditions, and people with fewer educational qualifications. Measures to support these endeavours include drawing on organisation-level tools such as the Race Equality Framework [59], ensuring that funding bids include costings for proper payment of participants, and inviting people from under-represented groups to collaborate on research design and recruitment.

Broadly speaking, the participants in the present research wanted routinely-collected mental health data to be put to use for the good of those living with mental illness. In practice, however, UK researchers’ ability to access these data is highly restricted and varies by location [8, 34]. Progress is being made via initiatives such as the SAIL databank in Wales [19], the Clinical Record Interactive Search (CRIS) in London [18], and DATAMIND, a UK-wide mental health data hub set up in 2021 [64]. With respect to the regulatory environment, the Goldacre Review, published in 2022, makes various recommendations for more streamlined sharing of health data in the future [53]. However, it remains unclear whether the proposed changes would be able to facilitate the time-sensitive cross-disciplinary data sharing necessary for highly effective mental health research [57]. Whilst safeguards are clearly important, it is essential that policy-makers do not let arbitrary restrictions limit the transformative potential of routinely-collected data for people living with mental illness.

## Conclusion

This interview study provided insight into how people with lived experience of mental illness feel about sharing routinely-collected mental health data. The findings bore similarities to previous work on the general public’s views on health data sharing, especially in the belief that health data should be used for societal good and protected by safeguards such as de-identification and secure data storage. However, the present findings also elucidated considerations around data sharing that are especially pertinent to those living with mental illness. On the whole, participants were driven by their own experiences with mental illness to support measures (such as secure data sharing) that could prevent other people suffering to the extent that they had done. They also frequently situated concerns around data misuse within the wider context of discrimination, envisioning or recalling incidents where information on their mental health status could be or had been used to discriminate against them. These findings broadly support researchers’ calls for more streamlined access to mental health data under appropriate conditions [34, 57]. Going forward, researchers and policy makers working in mental health data science should continue to strive for scientific and clinical progress whilst also acknowledging and seeking to reduce the stigma that threatens to hold this back.

### Electronic supplementary material

Below is the link to the electronic supplementary material.

Supplementary Material 1

## Data Availability

The dataset generated and analysed during the current study is not publicly available in order to protect participant privacy. An anonymised version of the dataset is available upon reasonable request from the corresponding author (Elizabeth Kirkham).
