# Supplementary material for: Views on sharing mental health data for research purposes: qualitative analysis of interviews with people with mental illness
Source: BMC Med Ethics. 2023 Nov 14;24:99. doi: 10.1186/s12910-023-00961-6 (PMC10648337; doi:10.1186/s12910-023-00961-6)
Supplement: Supplementary file 1 — Supplementary Material 1 [file 12910_2023_961_MOESM1_ESM.docx]

Appendix 1: Interview schedule

Research question: What do people **feel** about data sharing, and why do they feel that way?

In advance of interview:

- Send information sheet by email (or give them a physical copy if relevant)
- If participant hasn’t previously completed the survey then ask them to fill it in prior to the interview.

Prior to interview beginning:

- Make sure I have a copy of their mental illness responses to the survey, so I have an idea of their experience of mental illness and can reflect this information to them at the beginning.
- Fill in the required sections in the first question so they are relevant to the specific participant.

*Potential prompts (if participant requires more information) are written in blue italics.*

Definitions (if required during interview)

**NHS health records:** information about your physical and mental health which is recorded by a medical professional and stored by the NHS.

**Health data/health information:** information about your health which is collected by medical professionals.

**Physical health data:** weight, height, what conditions someone has been diagnosed with, what medicines they take, test results (such as blood test results), genetic information, GP notes, records of which services someone has used, medical images like X-rays.

**Mental health data:** what conditions someone has been diagnosed with, what medicines they take, scores on mental health assessment questionnaires, genetic information, GP notes, records of which services someone has used, medical images like MRI scans, notes taken during therapy sessions.

**Health research:** when health information is used to find out new things. It is normally carried out by universities, often in partnership with hospitals and sometimes with companies that make medicines and medical devices (e.g. pacemakers). Examples of health research might include looking at the long-term effects of taking vitamin supplements, or understanding how having a parent with dementia changes your chances of also having dementia.

Opening script

[Brief chat – what they did at the weekend/any plans for the weekend?]

As you know, my name is Lizzy and I’m a researcher working at the University of Edinburgh. I really appreciate you taking the time to participate in this interview. Today I’m going to ask you some questions about how you feeling about sharing NHS health records for research purposes. The background to today’s questions was described in detail on the information sheet I sent you by email. Would you like me to go over the information sheet?

Is it ok with you if I record the interview?

If yes: Great, I’ll let you know when I turn the recorder on.

*If participant queries recording: The recording, and the transcription of the recording, will be kept secure in the University of Edinburgh’s storage facilities. The files will be stored without your name attached, and I will not use your real name when I talk or write about the interviews later on.*

When talking about mental health I usually use the terms “mental illness” and “mental health condition”. Is this ok with you or would you prefer if I used different wording?

If you need a break at any time, just let me know. If there is anything you don’t want to talk about, or if you’d rather not answer a specific question, just say you’d rather not talk about it and I will move on. I am interested in your thoughts and opinions – there are no right or wrong answers. Please try to answer in as much detail as possible.

Do you have any questions to ask me before we start?

[Pause]

Are you ok to begin?

Great, I’m switching on the recorder now. *Remember to double check that it’s recording.*

1. We’re going to start by briefly reviewing your experiences with mental health. I realise we could talk about this for ages but I just want to check a few key details, so it would be great if we could keep this section to about five minutes. So, having had a look at your survey answers I can see that you’ve had experience with [X conditions, Q4.8], with [X condition, Q4.10] being particularly significant.

When you filled in the survey you were [select one before starting interview]:

1. not receiving NHS treatment for mental illness other than prescribed medication.
2. not receiving NHS treatment for mental illness.
3. receiving NHS treatment for mental illness, including prescribed medication.
4. receiving NHS treatment for mental illness but not taking any prescribed medication.

- Is this information still correct?
- Would you say your mental illness is something that comes and goes or something that has been or was with you for a long time?
  - *Some people have episodes where they are very unwell, and then times when their mental health is fairly good. Other people have mental health conditions that last for many years without much change.*
- Have you received any mental health support over the years? OR Is the treatment you’re receiving at the moment the first time that you’ve received mental health treatment, or have you received other treatment over the years?
  - [if so] Were you pleased with that treatment?
- How is your mental health at the moment?
  - *Compared to what is normal for you, how would you say you’re doing today in terms of your mental health?*
- Before we move on to the questions about data sharing, is there anything else that you want to highlight about your experiences with mental illness?
  - *Anything especially significant that comes to mind when you think about your mental health, or any thoughts or experiences that you feel have had an effect on how you think about mental health information that the NHS holds about you.*

1. The NHS holds information about you within your health records.

- What information do you think they hold?
  - *Information about your health that is recorded by medical professionals and stored by the NHS.*
  - *What do you think you would find if you looked inside your health records? What information do you think medical professionals might write down or store in your health records?*
  - [If not covered by participant] and do you think they hold any mental health data specifically?
  - *Mental health data might include what diagnoses you have received, what medicines you’re taking, images from brain scans, notes from therapists, notes from your GP, genetic information, and so on.*
- How does it make you feel to think about this topic – to think about the information they hold about you?
  - *Do you feel like this information is important? Do you generally feel positive or negative, or neither, when you think about these records?*

1. Imagine a researcher at a university wanted to access the information that is held in your NHS health records for a study they were carrying out. How would you feel about that?

- *[If query what information] Any information collected about you by a health professional. That could include information about your health conditions, therapist notes, medications you’re taking, brain scans, X-rays, blood test results, genetic information, and so on.*
- [If not covered] Why would you feel that way?

[Just to let you know, we’re about half way through the interview now.]

Concerns

- Would you have any concerns about a researcher accessing your NHS health records for their study? OR Thinking about the concerns that you have about a researcher accessing your NHS health records for their study…
- Are there any safeguards that could be put in place that would make you feel less concerned about a researcher accessing your NHS health records? OR [if not concerned] Can you think of anything that could be put in place that would give you additional confidence in how a researcher might use the information in your NHS health records?
  - *Name not connected to the data; researcher could only access files containing lots of people’s information, and not individual records; any researcher working with the information would need to work with an advisory group containing people who had experienced the health condition that the research was about; the researcher would have to pass a training course before being able to access the information, opt in or opt out.*
- [If not specified already] Why would this make you feel less concerned/give you additional confidence?

Use of mental health data

- What is the worst case scenario you could imagine happening as a result of your NHS data being shared with researchers?
- What is the best case scenario you could imagine happening as a result of your NHS data being shared with researchers?
- What do you think mental health data should be used for, if anything?
  - *To find out more about my mental health condition, find out about connections between mental and physical health, learn about the genetics of mental health conditions, find new treatments, reduce stigma, help prevent people from developing mental illness.*
- Why do you think mental health data should be used for this purpose/these purposes?

1. Now I’d like to ask about the types of health information a researcher might want to access for their study.

- Are there any types of health information that you think should be out of bounds for researchers?
  - *Information that a medical professional has written down or recorded about you. Types of health information: what you’ve been diagnosed with, what medicines you take, blood test results, brain scans, X-rays, therapy notes, GP notes.*
  - [if required] Why do you think this information should be out of bounds?
- Which kinds of health information, if any, do you think it would be important for researchers to be able to access?
  - *Information that a medical professional has written down or recorded about you. Types of health information: what you’ve been diagnosed with, what medicines you take, blood test results, brain scans, X-rays, therapy notes, GP notes.*
  - [If required] And which kinds of mental health data, if any, should they be able to access?
  - Why do you think researchers should be able to access this information?
- Imagine that a researcher was able to access information about your physical health, but not about your mental health. Would this affect how you felt about your health information being shared with researchers?
  - [if required] why would/wouldn’t this change how you felt?
- As well as health information, there are other sources of information about you which a researcher might want to access. This might include things like school attendance records, data from wearable fitness devices, social media posts, financial or employment records, whether you are married, and so on. How would you feel about a researcher connecting up these other types of information with information about your physical or mental health?

Closing

Thank you for sharing your experiences with me today. Do you have anything that you want to ask, or is there anything that I didn’t ask that you’d like to talk about?

Finally, I might use anonymous quotes from this interview when I’m writing about my findings. It won’t be possible for anyone to identify you from any quotes I use. Would you like to pick a made-up name for me to use if I include any quotes from your interview?

[pause]

Thank you again, it’s been great to talk to you. I will email over the debrief sheet which has more information about the project.

After interview, email debrief sheet.
